# Risk Factors for SARS among Persons without Known Contact with SARS Patients, Beijing, China

**DOI:** 10.3201/eid1002.030730

**Published:** 2004-02

**Authors:** Jiang Wu, Fujie Xu, Weigong Zhou, Daniel R. Feikin, Chang-Ying Lin, Xiong He, Zonghan Zhu, Wannian Liang, Daniel P. Chin, Anne Schuchat

**Affiliations:** * Beijing Center for Disease Prevention and Control, Beijing, China; † World Health Organization–China Office, Beijing, China; ‡ Centers for Disease Control and Prevention, Atlanta, Georgia, USA; § Beijing Municipal Health Bureau, Beijing, China; ¶ Beijing Joint SARS Expert Group, Beijing, China; 1 These authors served as temporary advisors to the World Health Organization–China Office SARS team

**Keywords:** SARS virus, case-control studies, risk factor, China, mask, disease transmission, disease outbreaks

## Abstract

Most cases of severe acute respiratory syndrome (SARS) have occurred in close contacts of SARS patients. However, in Beijing, a large proportion of SARS cases occurred in persons without such contact. We conducted a case-control study in Beijing that compared exposures of 94 unlinked, probable SARS patients with those of 281 community-based controls matched for age group and sex. Case-patients were more likely than controls to have chronic medical conditions or to have visited fever clinics (clinics at which possible SARS patients were separated from other patients), eaten outside the home, or taken taxis frequently. The use of masks was strongly protective. Among 31 case-patients for whom convalescent-phase (>21 days) sera were available, 26% had immunoglobulin G to SARS-associated coronavirus. Our finding that clinical SARS was associated with visits to fever clinics supports Beijing’s strategy of closing clinics with poor infection-control measures. Our finding that mask use lowered the risk for disease supports the community’s use of this strategy.

Severe acute respiratory syndrome (SARS) is a new disease caused by a previously unrecognized coronavirus (*1*,*2*). Investigations of SARS outbreaks in several countries suggest that the primary mode of transmission is close contact with a symptomatic patient. Indeed, most cases of SARS have occurred among persons who cared for or lived with someone with the disease, and this fact is reflected in the SARS case definition developed by the World Health Organization and in definitions developed by individual countries (*3*–*7*).

The SARS epidemic in Beijing, during which a total of 2,521 probable cases were reported from March through June 2003, was notable for its magnitude (*8*). Another distinguishing feature was the relatively high proportion of probable case-patients with no reported close contact with other SARS patients. Although the outbreaks in Hong Kong and Toronto were also large, most case-patients had healthcare-related or household links to other SARS patients (*4*,*9*). Beijing’s epidemic began with importations of SARS-associated coronavirus (SARS-CoV) in travelers returning from Guangdong Province and Hong Kong (*8*), and the first phase of the epidemic involved hospitalized patients, family members, and healthcare workers exposed to these travelers. During this period (March 8–April 3, 2003), almost all (96%) probable SARS patients reported close contact with a known SARS patient. However, during the peak of the epidemic (April 4–May 4), the percentage of probable SARS patients who reported no contact with another SARS patient and who were not healthcare workers rose to 42%; as the number of cases fell during the last part of the epidemic (May 5–June), this percentage increased to 65%. The reasons for these apparently unlinked SARS cases were unknown. Possible explanations included acquisition of disease from unrecognized sources in the community or healthcare setting, incomplete collection or recording of contact histories, and clinical illness that met the SARS case definition but was caused by etiologic agents other than SARS-CoV.

To evaluate these hypotheses, we conducted a matched case-control study during the Beijing outbreak among a sample of SARS patients who had no reported contact with other SARS patients.

## Methods

### Definitions

#### Probable Cases

Probable and suspected SARS cases were defined according to the China Ministry of Health’s definitions, which included clinical and epidemiologic components. The epidemiologic criteria changed during the course of the outbreak. Before April 3, 2003, only patients who had close contact with a known SARS patient or who had infected other persons could be diagnosed with SARS. From April 4 to May 3, the epidemiologic criteria were expanded to include persons with a history of visiting or residing in cities or areas where local transmission of SARS was occurring or with a history of contact with an outbreak or a healthcare facility. After May 3, Beijing was regarded as having local transmission of SARS, and visiting or residing in Beijing was considered sufficient to meet the epidemiologic criteria of the case definition. Laboratory testing for SARS-CoV was not part of the case definition.

### Close Contacts

Close contacts of SARS patients were defined as persons who shared meals, utensils, a residence, a hospital room, or a transportation vehicle with a suspected SARS patient or as persons who visited such a patient in a period beginning up to 14 days before the patient’s onset of symptoms. In addition, persons with potential contact with the bodily secretions of a SARS patient during the patient’s treatment or care were considered close contacts.

### Study Design

A matched case-control study design was used. Case-patients and controls were matched by sex and age group (<17, 18–25, 26–45, 46–64, and ≥65 years). The goal was to enroll 100 case-patients matched with three controls each, which, if one assumes an α of 0.05 and 80% power, would allow detection of an odds ratio of >2.3 for exposures observed in 15% of controls. For the analysis, we excluded all controls <14 years of age because of potential biases in comparing them with matched case-patients aged 14–17 years. In addition, case-patients who were reclassified as healthcare workers after interview were excluded along with their matched controls.

Case-patients were eligible for the study if they met the probable case definition and reported no close contact with any known probable or suspected SARS patients. Only patients whose hospitalization occurred after April 28, 2003, were included in the study. A list of patients admitted to the 16 designated SARS hospitals in Beijing was obtained periodically, and we called patients at the hospital ward or their homes (after discharge) to invite them to participate. The latest date of hospitalization included in our study was June 9, 2003. Case interviews were completed June 3–16.

We selected controls by sequential digit dialing, using the case-patient’s home telephone number as the index number. The last digit was added to or subtracted from by one digit in an alternating sequence until three controls matched by sex and age group were enrolled. Telephone prefixes are geographically clustered in Beijing, so this strategy was intended to provide neighborhood matching. Only one control was selected for each number dialed. Control interviews were completed by July 4.

### Data Collection

Data from case-patients were collected in person or by telephone, by using a standardized questionnaire. Information was collected on potential risk factors for, or exposures to, SARS-CoV infection (such as having a chronic disease or visiting a healthcare facility), personal hygiene (such as washing hands), and the use of masks. The period of inquiry was the 2 weeks before the patient’s onset of symptoms. For case-patients who reported visiting hospitals during the period of interest, a supplemental questionnaire was developed to collect detailed information on reasons for the visits and the hospitals and departments visited. Controls were interviewed by telephone and were queried about a reference period corresponding to the same 2-week period as the matched case.

Trained staff from the Beijing Center for Disease Prevention and Control interviewed all case-patients and approximately half of the controls. To accelerate enrollment, we used a commercial contractor to interview the remaining controls; the contractor received interviewer training by study staff before beginning the interviews. For quality control purposes, 10% of the contractor-interviewed controls were interviewed twice.

### Laboratory Tests

Case-patients were asked to come to Beijing Center for Disease Prevention and Control so that a 5-mL blood specimen could be obtained. Blood samples were centrifuged, and serum samples were refrigerated at 4°C. Sera were tested at the Beijing Center for Disease Prevention and Control for immunoglobulin (Ig) G antibody to SARS-CoV, by using an enzyme-linked immunosorbent assay kit (Beijing Huada GBI Biotechnology Co. Ltd., Beijing). All serum samples were obtained >21 days after illness onset, and 80% of them were obtained 76–106 days after onset of symptoms.

### Statistical Analyses

Matched univariate and multivariate analyses were conducted by conditional logistic regression. The PHREG procedure in SAS version 8 (SAS, Cary, NC) was used, with case status as the dependent variable. Factors associated with p values of <0.15 on univariate analysis were included in multivariable models. Collinearity and pair-wise interactions were evaluated for all variables in the final model.

## Results

A total of 373 patients were called from the master list until 100 were interviewed. Among patients who could be reached, the refusal rate was approximately 50%. The most frequent reasons for refusal were “tired of being interviewed” and being reluctant to disclose any personal information for fear of stigma and discrimination. Patients who agreed to participate in the study were similar in terms of age, sex, and temperature (on clinic presentation) to all probable SARS case-patients without a history of contact with another SARS patient (n = 1,091). Seven controls were excluded because they were <14 years of age, which resulted in the elimination of two matched sets. Four matched sets were also excluded because the case-patient was subsequently reclassified as a healthcare worker. A total of 94 case-patients and 281 matched controls were included in the final analyses.

Male patients accounted for 50% of case-patients. The median age was 29 years (range 14–84) for case-patients and 31 years (range 14–82) for controls. In univariate analyses, several health-related risk factors were significantly associated with an increased risk for clinically diagnosed SARS, including having visited any fever clinic (clinics established to separate patients who might have SARS from other persons being evaluated in emergency rooms or outpatient clinics) or any hospital or having a preexisting chronic disease, such as diabetes (Table 1). Eating out more than once a week and using several types of transportation, including taking a taxi or bus at least once a week, were associated with SARS (Table 1). Having visited a farmer’s market, wearing a mask when going out, and washing hands when returning home were protective factors. Factors that were not associated with SARS included visiting a school or university, participating in large social gatherings outside the home, having mice or cockroaches in the home, and having stayed home from work or school. No case-patients or controls reported having traveled to SARS-affected areas, such as Guangdong, Hong Kong, or Toronto.

**Table 1:** Selected potential risk and protective factors among cases and matched controls during the 2 weeks before the case-patient’s onset of SARS-related symptoms, Beijing, 2003^a^

Potential risk or protective factor for SARS	% of cases with factor N=94	% of controls with factor N = 281	Matched OR (95% CI) ^b^	p value
**Healthcare related**				
Visited any hospital	30	10	3.6 (2.0 to 6.5)	<0.001
Visited any fever clinic^c^	15	1	13.4 (3.8 to 46.7)	<0.001
Having any chronic disease^d^	19	7	4.1 (1.8 to 9.3)	<0.001
**Community related**				
Visited any school or college	14	16	0.8 (0.4 to 1.6)	0.52
Visited any quarantine site	2	2	1.2 (0.2 to 6.2)	0.83
Attended any social gathering^e^	7	10	0.8 (0.3 to 1.8)	0.52
Visited any movie theater, concert hall, or indoor gym	2	4	0.6 (0.1 to 2.8)	0.48
Visited any farmer’s market	23	37	0.5 (0.3 to 0.9)	0.01
Eating out				
Never	62	70	Reference	
Once a week	14	15	1.2 (0.6 to 2.4)	0.67
More than once a week	24	15	2.3 (1.2 to 4.5)	0.01
Riding a bus				
Never	62	73	Reference	
Once a week	13	7	2.3 (1.0 to 5.2)	0.04
More than once a week	25	19	1.7 (0.9 to 3.1)	0.08
Taking a taxi				
Never	80	79	Reference	
Once a week	7	16	0.4 (0.2 to 1.0)	0.05
More than once a week	13	4	3.2 (1.3 to 8.0)	0.01
Taking the subway				
Never	88	91	Reference	
Once a week	1	4	0.3 (0.0 to 2.3)	0.25
More than once a week	11	5	2.5 (1.0 to 6.6)	0.06
**Home related**				
Did not go to work/attend school	39	40	1.0 (0.6 to 1.6)	0.90
Had a pet	12^f^	20	0.5 (0.2 to 1.1)	0.08
Home infested by rats or mice	10	6	1.6 (0.7 to 3.9)	0.28
Home infested by cockroaches	16	15	1.1 (0.6 to 2.0)	0.87
**Behavior related**				
Wore a mask when going out				
Never	46	27	Reference	
Sometimes	27	30	0.5 (0.2 to 0.9)	0.02
Always	27	43	0.3 (0.2 to 0.6)	<0.001
Always washed hands before eating	83	89	0.6 (0.3 to 1.1)	0.11
Always washed hands after using restrooms	88	93	0.5 (0.2 to 1.2)	0.10
Always washed hands after returning home	78	90	0.3 (0.2 to 0.7)	0.003

^a^OR, odds ratio; CI, confidence interval; SARS, severe acute respiratory syndrome. ^b^Determined by use of conditional logistic regression. Exposures refer to the 2 weeks before symptom onset for cases and the same 2-week period for matched controls. ^c^Fever clinics were established for triage of patients who might have SARS to separate them from other persons being evaluated in emergency rooms or outpatient clinics. ^d^Includes diabetes, cancer, immunosuppressive treatment, and other. ^e^A gathering of >10 persons for a party or other social event. ^f^Pets reported by case-patients included dogs (3 cases), cats (3 cases), fish (1 case), and pigeons (1 case).

Factors associated with SARS in multivariable analysis are presented in Table 2. After other factors were controlled for, visiting a fever clinic and having a chronic medical condition remained significantly associated with a risk for SARS. After other variables were adjusted for, having visited a hospital was not associated with acquiring SARS. Other factors associated with an increased risk for SARS were eating outside the home and taking taxis more than once a week. Always wearing a mask when going out was associated with a 70% reduction in risk compared with never wearing a mask. Wearing a mask intermittently was associated with a smaller yet significant reduction in risk. Going to the farmer’s market and owning a pet were both protective factors.

**Table 2:** Factors significantly associated with acquisition of clinically diagnosed SARS in multivariate analysis^a^

Potential risk or protective factor for SARS	Matched OR (95% CI)^b^	p value
**Healthcare related**		
Visited any fever clinic^c^	12.7 (3.1 to 52.0)	<0.001
Having any chronic disease^d^	4.8 (1.7 to 13.2)	0.002
Visited any farmer’s market	0.4 (0.2 to 0.8)	0.01
Eating out		
Never	Reference	
Once a week	1.6 (0.7 to 3.8)	0.3
More than once a week	3.1 (1.2 to 7.7)	0.02
Taking a taxi		
Never	Reference	
Once a week	0.2 (0.1 to 0.8)	0.02
More than once a week	3.0 (0.9 to 10.3)	0.07
Had a pet	0.4 (0.2 to 0.9)	0.03
Wore a mask when going out		
Never	Reference	
Sometimes	0.4 (0.2 to 0.9)	0.03
Always	0.3 (0.1 to 0.6)	0.002

^a^OR, odds ratio; CI, confidence interval; SARS, severe acute respiratory syndrome. ^b^Fever clinics were established for triage of patients who might have SARS to separate them from other persons being evaluated in emergency rooms or outpatient clinics.

As of August 28, 2003, a total of 31 blood specimens had been tested for IgG to SARS-CoV, and 8 (26%) were positive. Of the eight seropositive case-patients, three had not visited a hospital or fever clinic in the 2 weeks before becoming ill.

## Discussion

SARS-CoV transmission is now understood to involve close contact of symptomatic patients with others. Surveillance and case management in most parts of the world have focused on patients with clinically compatible illness who had had exposure to another SARS patient or had traveled to an affected area. Once SARS was recognized as widespread in Beijing hospitals, respiratory illness in any Beijing resident raised suspicion of SARS, and health authorities urged a low threshold for consideration of SARS to institute patient isolation, case reporting, and contact tracing. In the Beijing outbreak, the large number of patients who were diagnosed with probable SARS without a contact history led to concerns that overdiagnosis was occurring or, alternatively, that unrecognized sources of transmission existed in the community. Our study suggests that both factors were involved.

Thirty percent of case-patients in this study had a history of visiting a hospital in the 2 weeks before onset of SARS. By univariate analysis, persons with SARS were more than three times as likely as age- and sex-matched controls to have visited hospitals. After other factors, including the presence of chronic medical conditions, were controlled for, visiting a hospital was not independently associated with a higher risk for clinical SARS. The frequency of a history of hospital exposure among our case-patients was consistent with the epidemiology of SARS observed in other major outbreaks, where hospitals served as important amplifiers of transmission. Instituting effective infection-control measures in healthcare settings is the most critical step in controlling the spread of SARS.

Fever clinics were established in Beijing for triage of patients who might have SARS to separate them from other persons being evaluated in emergency rooms or outpatient clinics. Our study found that visiting a fever clinic was a very strong risk factor for SARS. Through a follow-up questionnaire administered to patients who reported having visited hospitals or clinics, we attempted to ensure that the reported visits were for reasons other than the first symptoms of the SARS illness. Our finding that visiting fever clinics increased the risk for probable SARS infection confirms the suspicions of public health authorities that, early in the epidemic response, some fever clinics had not implemented appropriate isolation and triage procedures and supports the public health decision to close dozens of problematic fever clinics and enhance infection-control measures at the 66 clinics that remained open.

In this investigation, persons with chronic medical conditions also had a significantly higher risk of clinical SARS developing. A disproportionate occurrence of the disease in persons who are elderly or who have a chronic disease was noted in other SARS outbreaks, but whether these factors were just markers for persons likely to have nosocomial exposure to other SARS patients was unclear. Our study found that the SARS risk associated with chronic disease was independent of recent exposure to healthcare facilities and suggests that, as is the case for other types of pneumonia (*10*,*11*), persons with chronic medical conditions are more vulnerable to clinically defined SARS. We had insufficient numbers of laboratory-confirmed cases to verify that this finding was specific for SARS-CoV infection.

Because a considerable proportion of SARS cases were reported in persons without a history of contact with another SARS patient and without exposure to healthcare facilities, we sought to identify unrecognized sources of community transmission that might help target control strategies and clarify whether widespread community transmission was indeed occurring. We found that certain community exposures were significantly more common among case-patients than controls, including eating out or taking taxis frequently. By univariate analysis, use of other common transport (e.g., buses, subways) was also associated with a risk for SARS. At least one well-publicized case of SARS in Beijing occurred in a taxi driver (*12*), but an increased risk among passengers had not previously been documented. Our findings regarding use of transportation bordered on statistical significance and will require validation by other studies.

We also used this investigation to quantify the impact of behaviors (i.e., mask wearing, handwashing) that were promoted to reduce the risk for SARS. Wearing masks outside the home in a reference period corresponding to the 2 weeks before symptom onset for cases was significantly protective against clinical SARS. Supporting the validity of this finding, there was a dose-response effect: by multivariable analysis, persons who always wore masks had a 70% lower risk of being diagnosed with clinical SARS compared with those who never wore masks, and persons with intermittent mask use had a 60% lower risk. Many persons who wore masks in the community did not use N-95 or similar highly efficient filtration devices, which have been recommended for use in the hospital setting. We sought details on the type of masks used but were unable to evaluate the protective efficacy for different mask types. We also were not able to differentiate protective efficacy for SARS-CoV versus efficacy against other pneumonia causes that met the clinical case definition.

Handwashing has been recommended to prevent SARS and other respiratory and diarrheal infections in which contact is an important mode of transmission. We found that consistently washing hands upon returning home was associated with a reduced risk for clinical SARS by univariate but not multivariate analysis. However, self-reported handwashing practices may be particularly prone to misclassification because respondents might provide the answer they believe is expected of them.

We also explored the role of domestic animals in relation to SARS infection among persons without contact with another SARS patient. An animal source for the origin of SARS-CoV in humans is suspected (*13*), and, using polymerase chain reaction, investigators identified SARS-CoV in household pets and cockroaches at the Amoy Gardens apartments in Hong Kong (*14*). Thus, we wondered whether certain household pets or rodents might be perpetuating disease-transmission cycles. One investigator recently hypothesized that a rodent vector may have amplified transmission of SARS at Amoy Gardens (*15*). In addition, rumors circulating during the Beijing SARS outbreak led to some calls for banning household pets or restricting them from common areas. We sought evidence to address this community fear and found that household rodents and cockroaches were not associated with a risk for clinical SARS. We also found that persons with pets had a significantly lower risk for clinical SARS. This finding might have occurred by chance or may be confounded by another factor more directly related to pneumonia. However, controls with pets might possibly have had exposure to other animal coronaviruses that provided cross-reacting antibody to the SARS-CoV. Of note, other investigators found IgG to SARS-CoV was common among animal traders in Guangdong (*16*), yet disease did not occur in this population, a finding consistent with the hypothesis that cross-reacting antibodies to a closely related virus may have protected these workers.

Another unexpected finding was that visiting a farmer’s market was associated with a reduced risk for clinical SARS. Nevertheless, concern that farmers represented travelers from other provinces and that markets were crowded settings prompted us to ask about this exposure as a possible risk factor. Accounting for an association with lower risk is challenging. As with ownership of pets, this finding may relate to unmeasured lifestyle factors more directly related to pneumonia risk.

Among authorities in Beijing, a leading hypothesis for the occurrence of clinical SARS among patients without known contact with another SARS patient was that overdiagnosis was occurring. We sought to determine the proportion of case-patients in this study who could be confirmed by convalescent-phase serologic tests to be infected with SARS-CoV; however, we obtained serum samples from an insufficient number of case-patients to analyze risk factors for laboratory-confirmed cases. Serologic testing for SARS may not be 100% sensitive, and the Huada test kit has had limited validation thus far. Nevertheless, a substantial portion of case-patients without contact with other SARS patients likely had pneumonia caused by pathogens other than SARS-CoV.

Certain limitations to this study should be mentioned. First, the study was conducted late in the Beijing epidemic, after patients had been hospitalized for several weeks, and the low participation rate might be attributable to patients having already been interviewed multiple times. Furthermore, recall bias might have influenced some of the factors we explored. Telephone-based public health studies were relatively new to Beijing, and the representativeness of our control population is not known. Because the rate of study participation by case-patients was not high, those who agreed to participate may have self-selected for unknown reasons that could have biased our findings. For instance, several patients responding to the open-ended comment section mentioned that they were certain their illness was “not SARS.” Relatively few patients agreed to convalescent-phase serologic testing, and those who did agree may have been more skeptical about the cause of their pneumonia than were others, which may have skewed the sample for which we have serologic results.

In conclusion, we identified several explanations for the occurrence of clinically defined SARS in persons without contact with another SARS patient during Beijing’s 2003 SARS epidemic. The nonspecific clinical definition for SARS led to reporting of many cases that were not confirmed to be caused by SARS-CoV. This apparent overdiagnosis probably helped ensure rapid control of the outbreak by introducing a wide net for contact tracing and patient isolation. Increased risk for clinically defined SARS was associated with attending fever clinics, having a chronic disease, and having certain community exposures. Consistent mask use lowered the risk for disease, thus providing some justification for the use of a strategy that was very popular in the general community. Our finding that pet owners had a lower risk for clinical SARS can help dispel fears that domestic pets were causing disease transmission in Beijing. Improved laboratory diagnostic tests (i.e., tests with high sensitivity early in the illness and with rapid turnaround) may eventually allow for more specific case reporting and management. Although human-to-human transmission of SARS has apparently been interrupted as of this writing, the factors associated with clinically defined SARS in this study may help target future efforts to control other respiratory infections, including pandemic influenza, and will provide valuable evidence for the control of SARS should the disease return.
